# Influence of external insert angle on structural deformation of long pipe shed with shallow hole

**DOI:** 10.1038/s41598-023-41321-z

**Published:** 2023-08-29

**Authors:** Chao Teng Jiang, Wei Mao, Yongkang Zhang, Xuejun Liu, Ruheiyan Muhemaier, Liangfu Xie

**Affiliations:** 1 College of Civil Engineering and Architecture, Xinjiang University, Urumqi, 830046 China; 2 Xinjiang Vocational and Technical College of Communications Xingjiang Urumqi, Urumqi, 831013 China; 3 CCFEB Civil Engineering Co., Ltd., Changsha, 410004 China; 4 Xinjiang Academy of Architectural Science (Limited Liability Company), Urumqi, 83002 China

**Keywords:** Engineering, Civil engineering

## Abstract

The long pipe shed advanced support has the characteristics of large construction difficulty, wide support range, and easy deviation. The difference in construction dip angle will produce a different plastic zone of surrounding rock and supporting effects, and the rationality of advanced support design plays an important role in the safety of tunnel opening excavation. Based on the Tianshan Tunnel project, this paper aims at the problems of the loose pebble layer structure, poor cohesion, strong permeability, shallow excavation is not easy to form a confined arch, and easy to causes surrounding rock deformation and failure. Combined with the new method theory, the finite difference software FLAC^3D^ was used to simulate seven excavation schemes of the shallow tunnel entrances. The mechanism and effect of advance support and the influence of construction angle on support effect are analyzed, and the simulation values are compared with the measured data. The results show that using long pipe shed advanced support can effectively reduce the disturbance of excavation to lose pebble rock mass and reduce the convergence value of surrounding rock. The plastic zone of surrounding rock produced by different angles can be divided into three parts. The structural stability difference of the C_1_ zone is small, and that of the C_3_ zone is large. The bending moment, shear force, and shape variables of the pipe shed decrease with the increase of the dip angle, while the axial force increases with the increase of the dip angle. According to the similarity between the simulated curve and the measured curve, the best supporting effect can be achieved when the dip angle of the pre-supporting pipe shed is set in the range of 0° ~ 3°.

## Introduction

Tianshan Region of Xinjiang has very complicated geological conditions, and complex engineering geology such as faults, broken surrounding rock, and water gushing is often encountered in the process of tunnel construction^1–3^. In engineering design, it is inevitable to encounter the construction of shallowly buried complex strata. As the first step of tunnel excavation, the tunnel entrance construction is related to the safety and quality of the whole tunnel project. For the construction of the Tianshan long tunnel, the construction of its entrance section is a shallowly buried pebble layer excavation. Due to the loose structure and poor cohesion of the pebble layer, it is difficult to arch the excavation face when the tunnel passes through the pebble layer, resulting in a large formation disturbance^4^. If the design scheme is not reasonable, the excavation of the entrance will cause excessive deformation of the surrounding rock, slope sliding, roof collapse, and other engineering disasters. Therefore, the design of a reasonable support scheme to control the deformation of surrounding rock is the top priority in the construction of shallow-buried pebble tunnels^5^.

The surrounding rock at the entrance section of a tunnel is generally shallowly buried, and its self-stabilization ability is poor. The traditional Austrian method only focuses on the excavation method, without controlling ahead of the face soil, and there is no pre-consolidation deformation during construction^6–8^. To better meet the requirements of complex geological tunnel construction, Pietro Lunardi^9^ proposed the ADECO-RS Approach based on the traditional Austrian method, which emphasizes improving the stability of the ahead-of-the-face soil and the tunnel lining through pre-reinforcement. This concept has great advantages in the construction of shallowly buried tunnels in gravel layers^10^. The ahead of the face support^11,12^ is commonly used in engineering cases to reinforce the surrounding rock and the tunnel lining, mainly through ahead-of-the-face anchor bolts or small guide tubes, ahead of the face pipe shed, grouting to reinforce the surrounding rock, or intercepting water. With the advancement of industrial technology, the construction of pipe shed grouting has developed rapidly and progressed. Liu^13^ combined with the case of a granite water inrush tunnel in Guangxi, the grouting effect was compared and summarized by using 25 grouting cycles and excavation operation, and the supporting effect under different grouting parameters was analyzed. According to different seepage environments, different selection criteria of grouting materials are proposed. Zhao^14^ according to the characteristics that the water-bearing sand layer is easy to cause large deformation of underground pipe corridor, a double-layer support system composed of a pipe shed and horizontal jet grouting pile is proposed. According to the stress characteristics of the upper beam on the elastic foundation, the stress model and analysis method of the structure are established. The feasibility of the double support reinforcement scheme is verified by comparing the simulated value with the measured value. In addition, many scholars^15–17^ have conducted extensive research on advanced support technology and its application in multi-engineering geology, and have achieved relevant research results. Although many scholars have studied and achieved a lot of results on the advanced support construction of long pipe sheds, due to their long size and the different angles of support, the support effect varies. There are still relatively few results on the impact of construction angles on the effect of advanced support. This paper relies on the construction background of the Tian Shan Extra Long Tunnel, simulates seven different excavation schemes, comprehensively analyzes the results from multiple angles with the theory of new integral law, and combines the on-site measured values to verify the simulation results to provide reference opinions for relevant projects in the future.

## Project profile

### Geology

**Figure 1:**
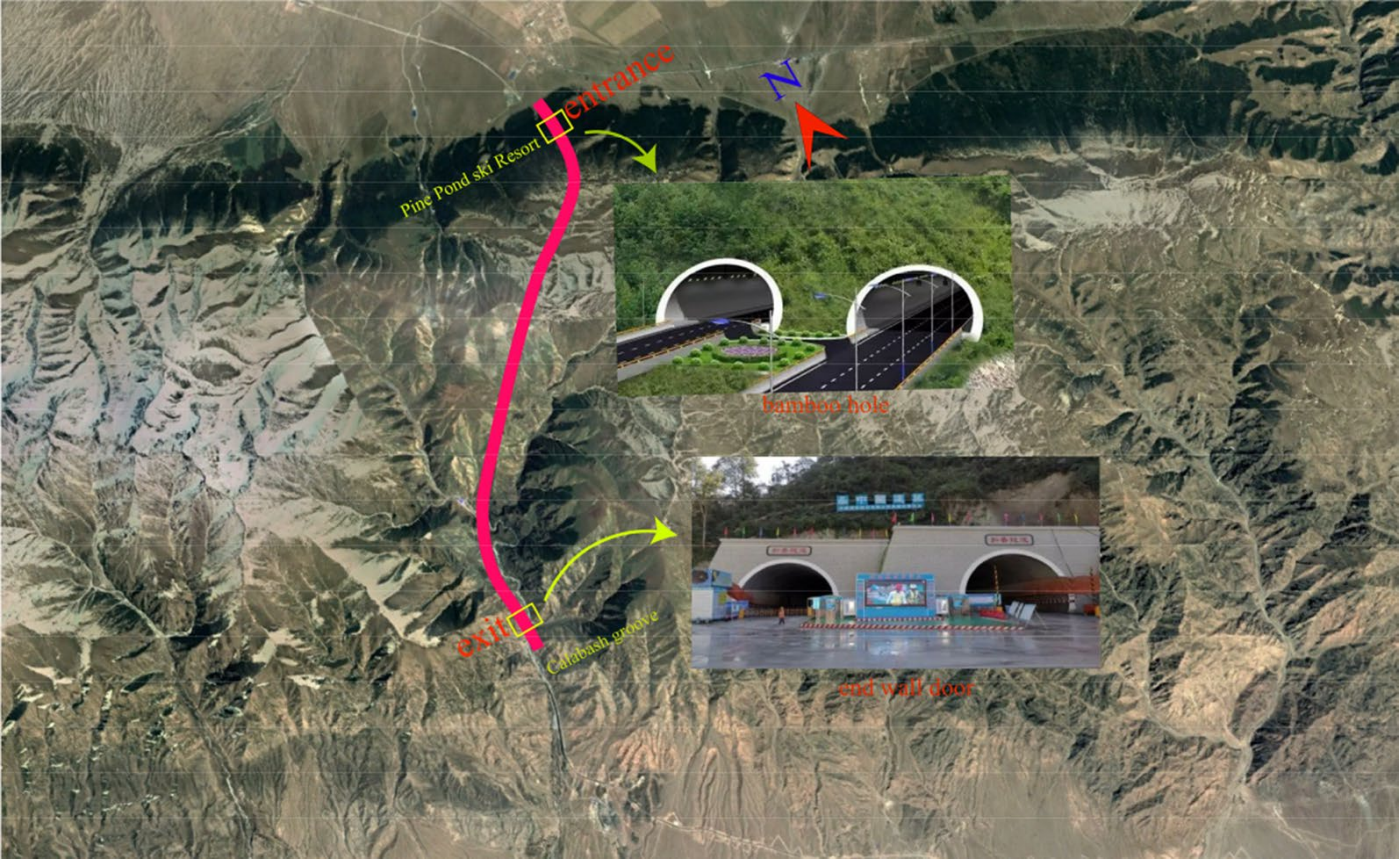
Tunnel azimuth map (Map download from the four-dimensional remote sensing cloud service platform https://siweiearth.com/sw-earth/home).

**Figure 2:**
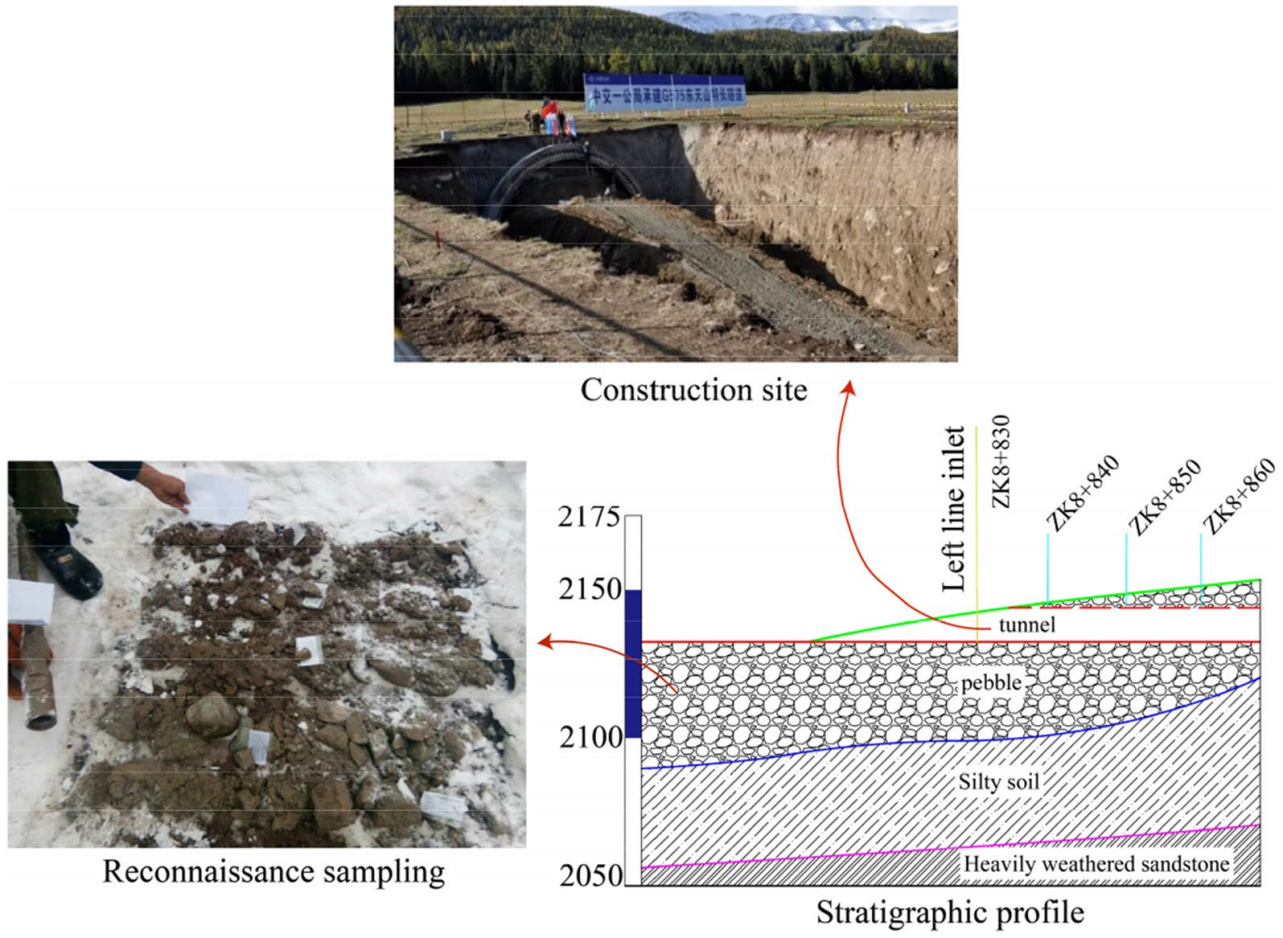
Stratigraphic profile.

G575 line Tianshan tunnel is separated from the left and right, the distance between the measured lines is 30 m, the length of the left hole is 11,870 m, the length of the right hole is 11,900 m, and the entrance of the tunnel adopts the bamboo cutting entrance (the tunnel door structure is in the form of bamboo cutting), and the exit of the tunnel adopts the end wall tunnel door (the tunnel door structure is simple and suitable for stable areas). Combined with the construction scheme, the excavation simulation of pile number ZK8 + 830 ~ ZK8 + 860 at the entrance of the tunnel left line is carried out. This section of tunnel is located in Hami Region of Xinjiang, China, which is located in the hinterland of Eurasia continent at mid-latitude. It is dry all the year round with little rain and obvious temperature difference. The entrance of the tunnel is located at the downstream side of the Pine Pond ski Resort in the north of Tianshan Mountain, and it is a gentle slope platform between the two gullies. The entrance elevation of the tunnel is about 2129 m, the topographic slope is 13°, and the top is steep and the bottom is slow. This section of the tunnel is covered by loose pebbles with a thickness of 38.4 m ~ 70 m. The underlying bedrock is late Pleistocene diluvial silty clay, and the surrounding rock itself has poor stability. According to the field investigation and sampling laboratory test, the internal friction angle of pebbles in this area is 30°, the internal cohesion is 10.18 kPa, and the soil layer is V grade surrounding rock weathering seriously. The overall orientation of the tunnel is shown in Fig. 1, the stratum profile of the tunnel is shown in Fig. 2, and the mechanical parameters of surrounding rock are shown in Table1.

**Table 1:** Physical parameters of soil layer.

Name	Unit weight/(kN m^−3^)	Elasticity modulus/MPa	Poisson's ratio	Internal friction angle/(°)	Cohesion/kPa
Pebble	21	35	0.2	30	10.18
Silty clay	19.5	30	0.25	28.2	26

### Hole excavation scheme

**Figure 3:**
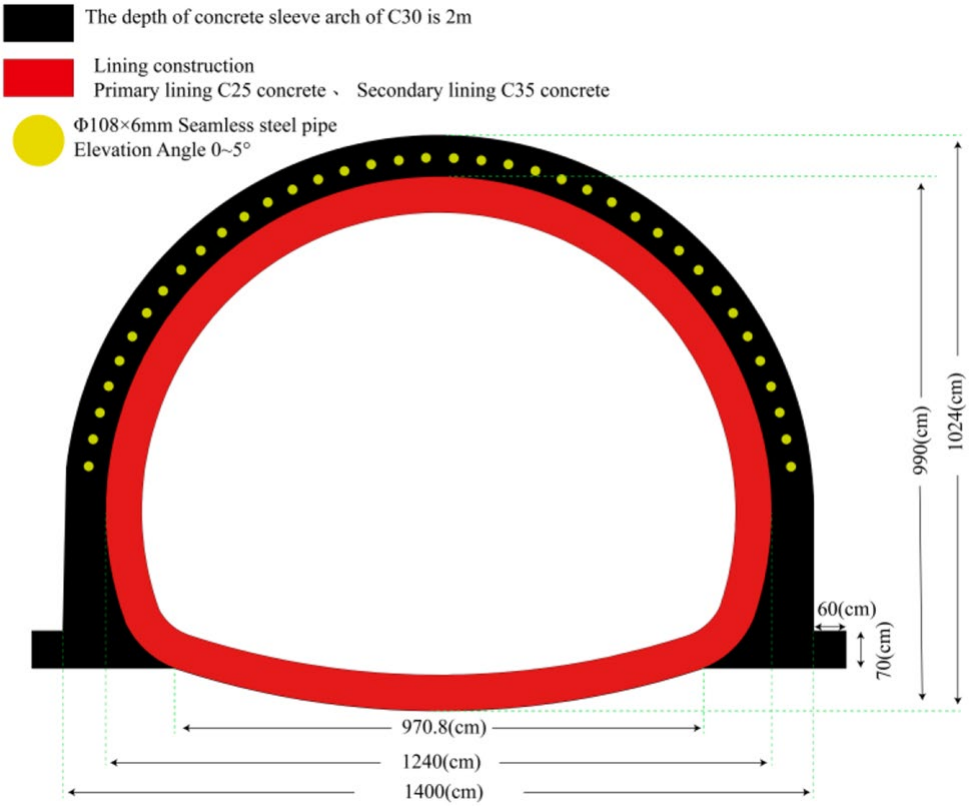
Design drawing of lining structure of opening section.

According to the survey data, the buried depth of the ZK8 + 830 ~ ZK8 + 860 section is 7 ~ 11 m, and the entrance section is a shallowly buried tunnel excavation. The construction of a pipe shed with arch sets is adopted. The total length of the pipe shed is 30 m, and the depth of the arch sets is 2 m. And the Φ133*4 mm guide tube is fixed in the sleeve arch. Pipe shed using Φ108*6 mm, adjacent steel pipe ring spacing is 30 cm. Combined with the new method, the excavation simulation of the opening was carried out. The section width of the excavation was 12.4 m and the height was 9.9 m. The excavation scheme was full-section excavation. The construction structure of the entrance is shown in Fig. 3, and the physical and mechanical parameters of the structure are shown in Table 2.

**Table 2:** Structural physical parameter.

Name	Bulk density/(kN m^−3^)	Modulus of elasticity/MPa	Poisson's ratio	Compressive yield stress/MPa	Tensile yield stress/MPa
Sheath arch	25	30,000	0.15	38.2	2.01
Primary lining	24	28,000	0.13	28.75	1.78
Secondary lining	25	30,000	0.15	38.2	2.01
Pipe shed	33	93,000	0.3		

## ADECO-RS approach stability theory

### ADECO-RS approach research theory

**Figure 4:**
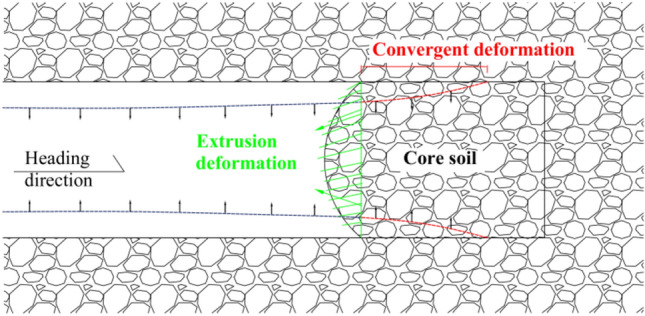
Deformation characteristics of the new Italian method of surrounding rock.

The basic principles of the ADECO-RS approach include excavation of the full section of the tunnel, reinforcement by using advanced support to reduce or avoid large deformation of surrounding rock, and short-distance support on the face of the face to reduce tunnel deformation. The study takes into account the relationship between the open face and the supporting structure, as well as the stress variation characteristics of the arch effect of the excavation section, and comprehensively analyzes the change law of surrounding rock after tunnel excavation. The principle of surrounding rock deformation characteristics of the new method is shown in Fig. 4.

**Figure 5:**
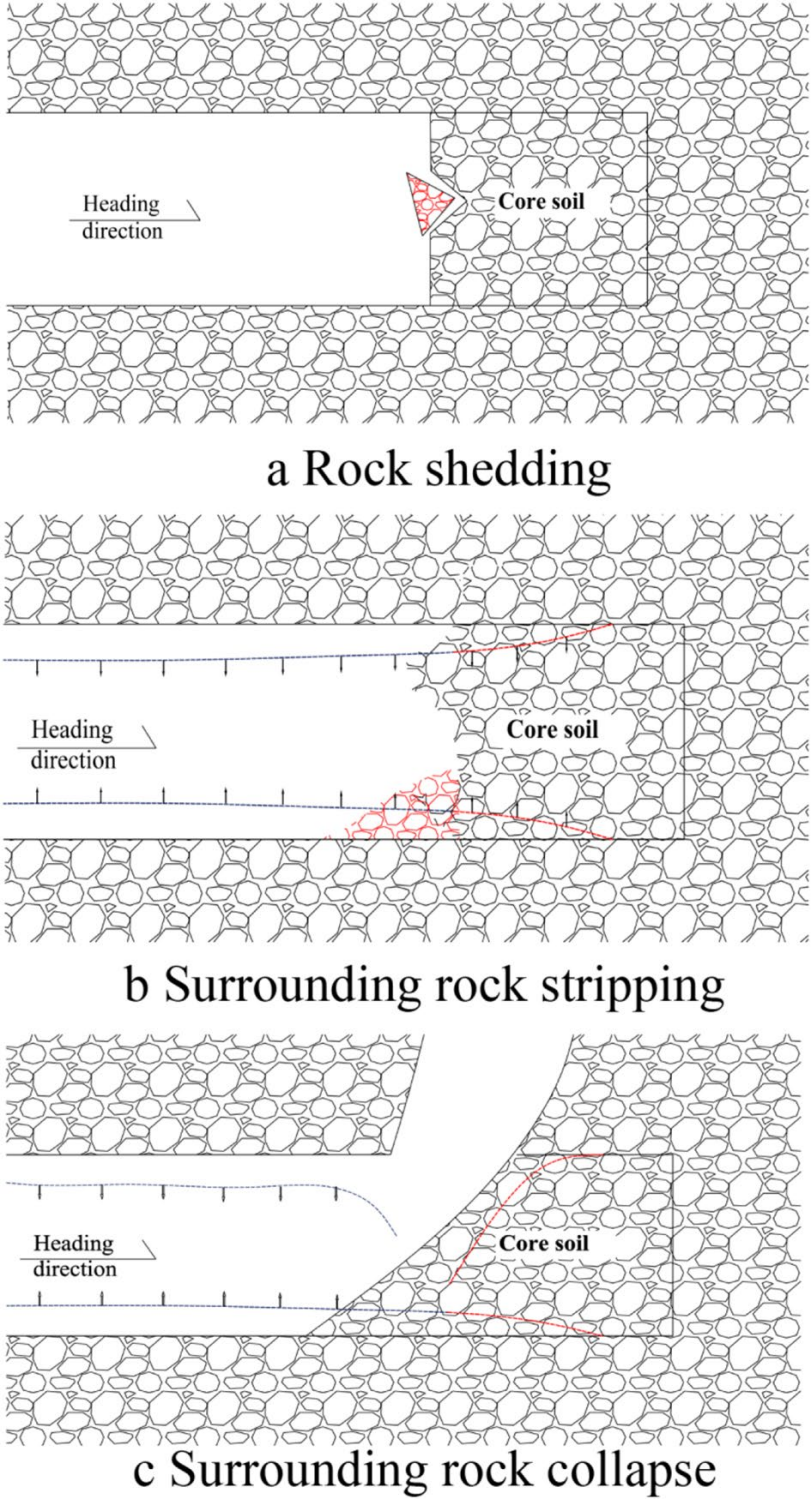
Deformation evolution of surrounding rock.

The main content of the ADECO-RS approach includes three parts: advanced core soil, extrusion deformation of the palm face, and pre-convergence deformation. The core idea is deformation stability during the excavation process. The deformation stability of the surrounding rock during tunnel excavation is related to the strength and stiffness of the advanced core soil. The surrounding rock structure of the pebble layer is loose and has low cohesive strength. If it cannot be controlled promptly on time during the excavation process, it is highly prone to engineering disasters, such as arch collapse, surrounding rock detachment, and large deformation of the surrounding rock. Combined with the new concept method, the three deformation evolution processes that are prone to occur during the excavation of the pebble layer are shown in Fig. 5.

**Figure 6:**
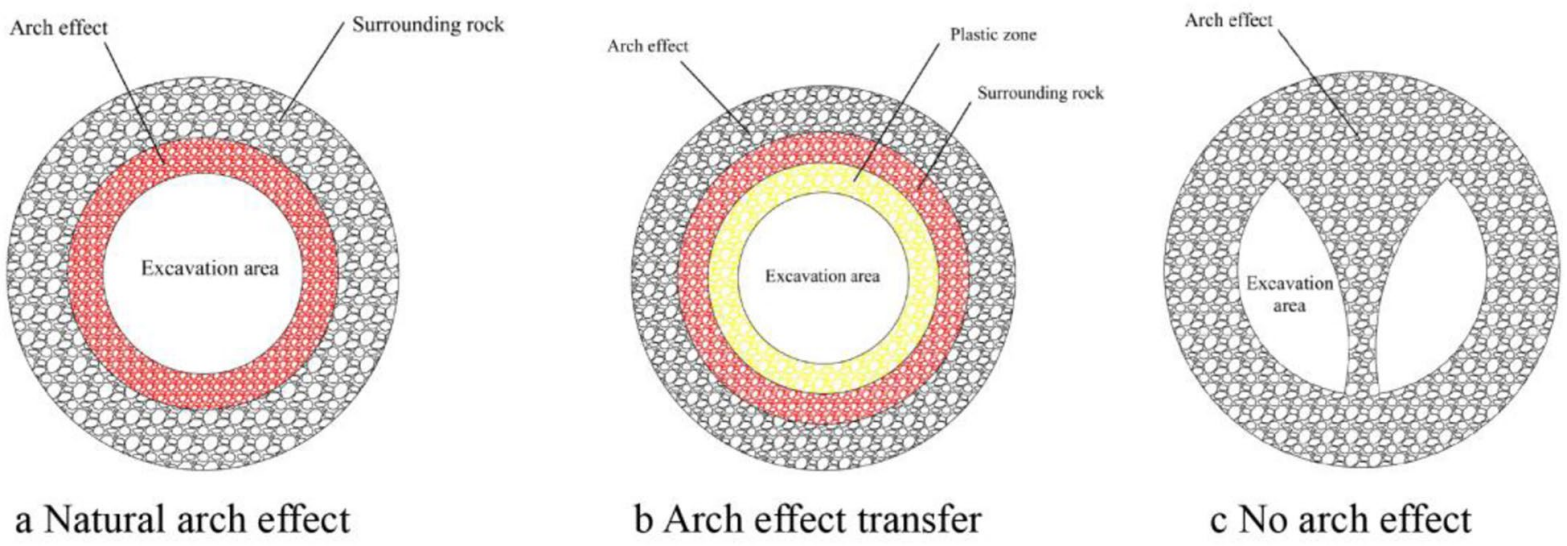
Arch effect diagram.

After the excavation of the surrounding rock of the tunnel contour is completed, a cavity is formed below the arch rock mass; Under the influence of gravity, the stress of the surrounding rock will continuously self-adjust, and the surrounding rock will converge and deform towards the free surface under the action of stress. The stress generated by the cavity in the surrounding rock mass outside the contour line will increase, which is called the arch effect. When the surrounding rock has high strength, good stability, and strong bearing capacity, its arch effect is close to the contour line of the cavity, called the natural arch effect (as shown in Fig. 6a). When the bearing capacity of the surrounding rock is slightly poor, the arch effect area will transfer along the contour line to the deep part of the surrounding rock, and the surrounding rock will form a plastic zone with poor stability, called the transfer arch effect (as shown in Fig. 6b). When the surrounding rock structure is loose and the bearing capacity is poor during excavation, there will be large-scale sliding on the face and collapse of the arch roof. At this time, the arch effect of the surrounding rock is difficult to form, which is called the noarch effect (as shown in Fig. 6c).

### Analysis of advanced supporting principle

**Figure 7:**
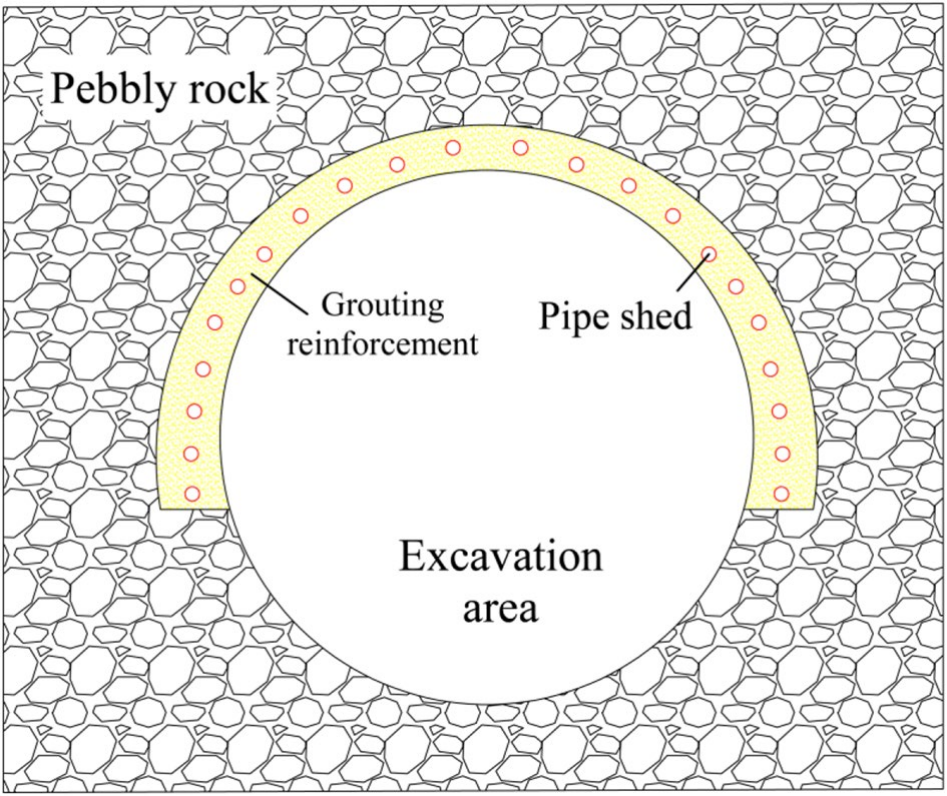
Over-supporting range map.

The structure of the pebble layer is loose and the cohesion is low, so it is not easy to form the arch effect during excavation, which leads to the large-scale sliding of the palm surface and the collapse of the arch roof. The protection range of advance support is generally set below the arch waist to increase the contact area with the contour line; Into the 30 m long pipe shed as a guide pipe to inject cement grout, and cement slurry from the pipe shed plum hole to the four sides and the surrounding rock solidification reaction to form a thickness of about 80 cm concrete structure. The pipe shed, concrete, and pebble surrounding rock together form a reinforced concrete component, forming a bearing arch above the tunnel contour line to ensure construction safety. The protection range of advanced support is shown in Fig. 7.

## Numerical test model component

### Model geometry size determination

**Figure 8:**
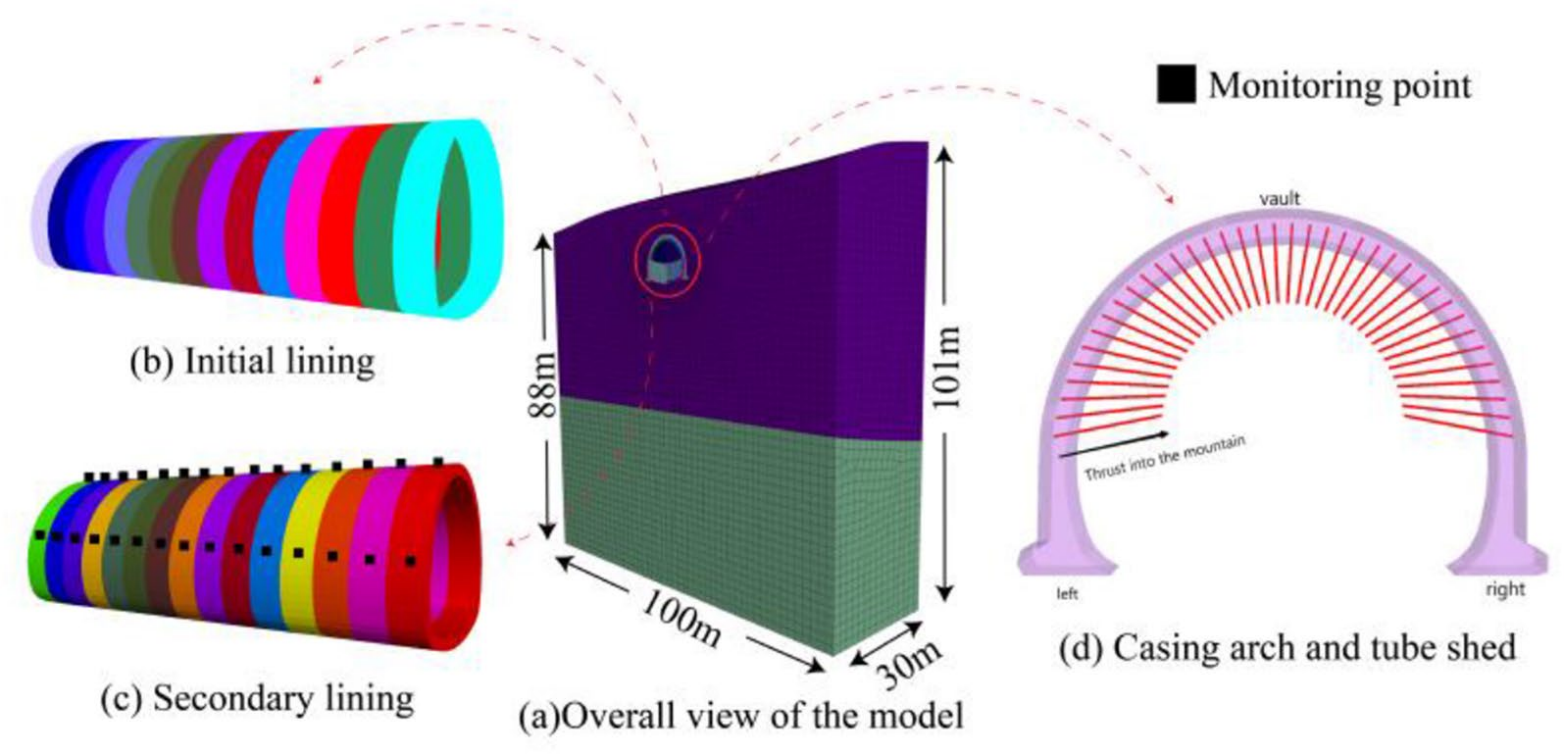
Model building diagram.

The surrounding rock with a depth of 30 m from the left opening of the Tianshan Tunnel was taken as the simulation object. The full section excavation was adopted, and the progress of each cycle was 2 m. A total of 15 sections were excavated. The pile number ZK8 + 830 at the entrance was selected as the analysis section (7 m buried depth), and the model was established according to the tunnel design drawing and geological survey data. The calculation range was selected to eliminate the influence of boundary effect along each direction of the tunnel diameter by 3 ~ 4 times, the simulated stratigraphic range 4 times the hole diameter (12.4 m) at both ends of the transverse is 49.6 m, and the width of the model is 100 m. The subsoil was 41 m (10.24 m), the total height of the front end of the model was 88 m, and the maximum height of the back end was 101 m. In the three-dimensional analysis, different constitutive models were assigned to the model. The soil layer adopted Mol–Coulomb constitutive model, the lining adopted the Elastic constitutive model, the initial lining adopted the shells element, and the ultra-long pipe shed support adopted the beam structural element. In the early stage, all the models adopted the Mol–Coulomb model, and the corresponding soil layer parameters were assigned. By modifying the constitutive model and material parameters, the lining structure can be distinguished from the soil layer. To improve the calculation accuracy, a fine mesh is used near the tunnel structure. In addition to the upper part of the model as the vertical load boundary, normal constraints are added to the remaining surface and the bottom surface, which imposes rotation constraints on the pipe shed structure. Since the excavation section of the tunnel entrance is shallow and there is no groundwater influence, only the dead weight stress is considered. The model establishment is shown in Fig. 8.

### Model scheme comparison

To better obtain the supporting effect of advanced support on shallowly buried tunnel excavation and the influence of the construction angle of pipe shed on structural deformation, seven different schemes were used for comparison and verification. The specific content of the simulation scheme is shown in Table 3.

**Table 3:** Comparison of simulation scenarios.

Scenario name	Whether there is advanced support or not	Pipe shed angle (°)	Grout diffusion radius (m)
Plan I	No		
Plan II	Yes	0	0.4
Plan III	Yes	1	0.4
Plan IV	Yes	2	0.4
Plan V	Yes	3	0.4
Plan VI	Yes	4	0.4
Plan VII	Yes	5	0.4

**Figure 9:**
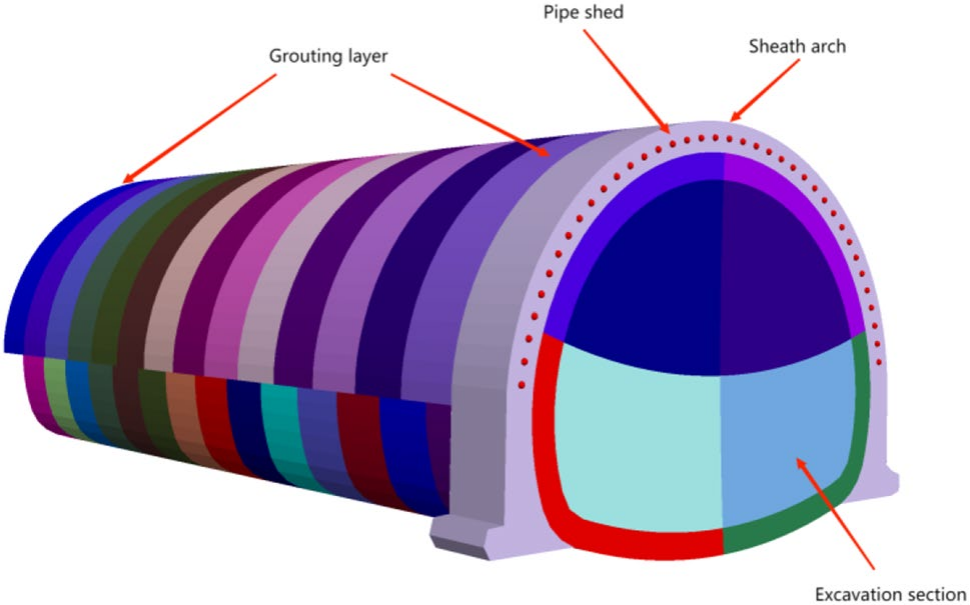
Over-supporting scheme.

**Figure 10:**
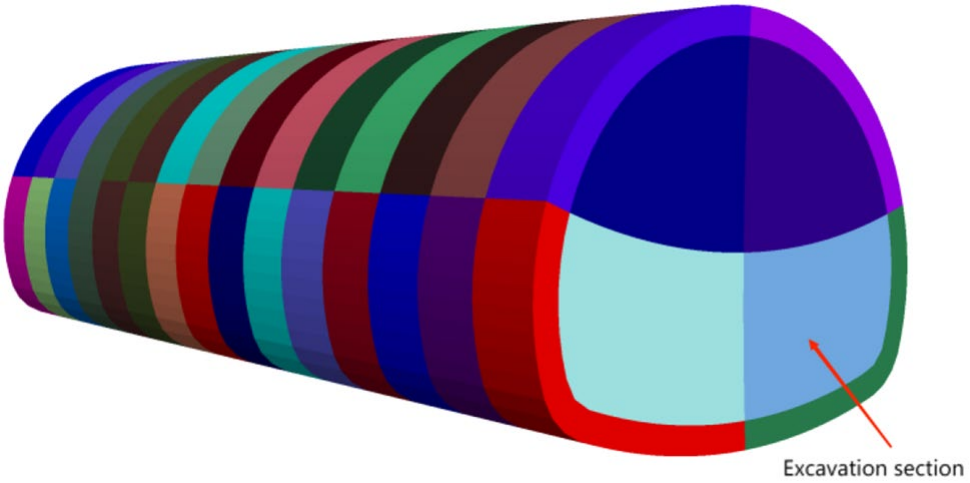
No over-support scheme.

**Figure 11:**
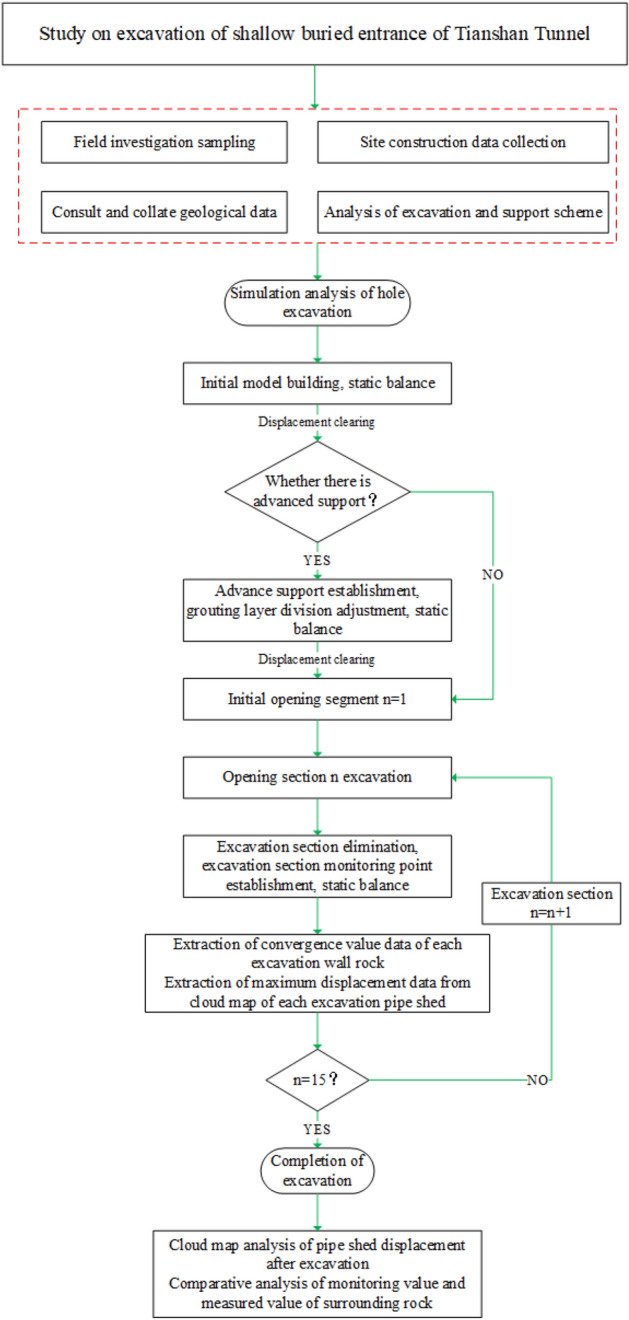
Study flow chart.

In order to ensure that the angle of the pipe shed simulation is accurate, the initial model is generated by geometric model in Midas gts NX software, and the accurate position of each beam unit is generated by modeling in FLAC^3D^. According to relevant literature^18^, the physical and mechanical parameters of surrounding rock will be improved differently after grouting reinforcement. The surrounding rock reinforced by grouting increases the original elastic modulus by 42 ~ 56%, the cohesion by 35 ~ 51%, and the friction angle by 2.3° ~ 3.4°. Due to the broken structure and poor lithology of the pebble layer, the transverse deformation and tensile strength changes of the surrounding rock after grouting reinforcement are not considered in this study. Combined with the geological survey data, the parameters of the rock mass inserted by beam unit are synthesized. In order to simulate the reinforcement effect of grouting, the elastic modulus and cohesion of the soil in the grouting layer (Fig. 9) are increased by 40% and the friction angle by 3° on the basis of the original parameters. Excavation for advanced support is shown in Fig. 10. The research flow chart is shown in Fig. 11.

## Analysis of numerical simulation results

### Comparison of pipe shed displacement

**Figure 12:**
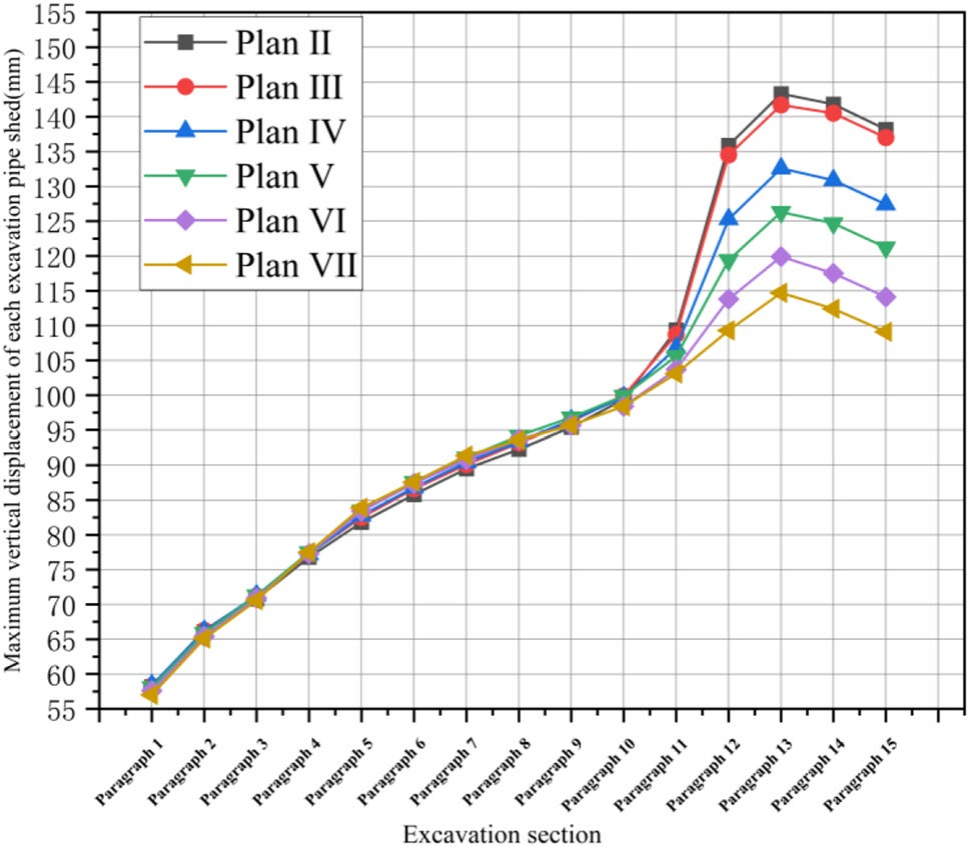
Comparison of maximum vertical displacement of pipe shed.

As shown in Fig. 11, after the completion of a section (2 m) of simulated excavation, the maximum subsidence displacement value generated by excavation disturbance in the displacement cloud map of pipe shed was recorded and aggregated into a table as shown in Fig. 12. According to the analysis of Fig. 12, after the excavation of the first nine sections of advance support, the vertical displacement gap corresponding to the pipe shed structure with different insertion angles is small; after the excavation of the tenth section, the vertical displacement gap gradually increases, the vertical displacement of the thirteenth section reaches the maximum value, and the subsidence of the pipe shed in the back excavation gradually decreases. In the comparison of six schemes, the vertical displacement of pipe shed in plan II is the largest, while that in plan VII is the smallest. In the maximum comparison in paragraph 13, plan VII was 21% less than plan II.

**Figure 13:**
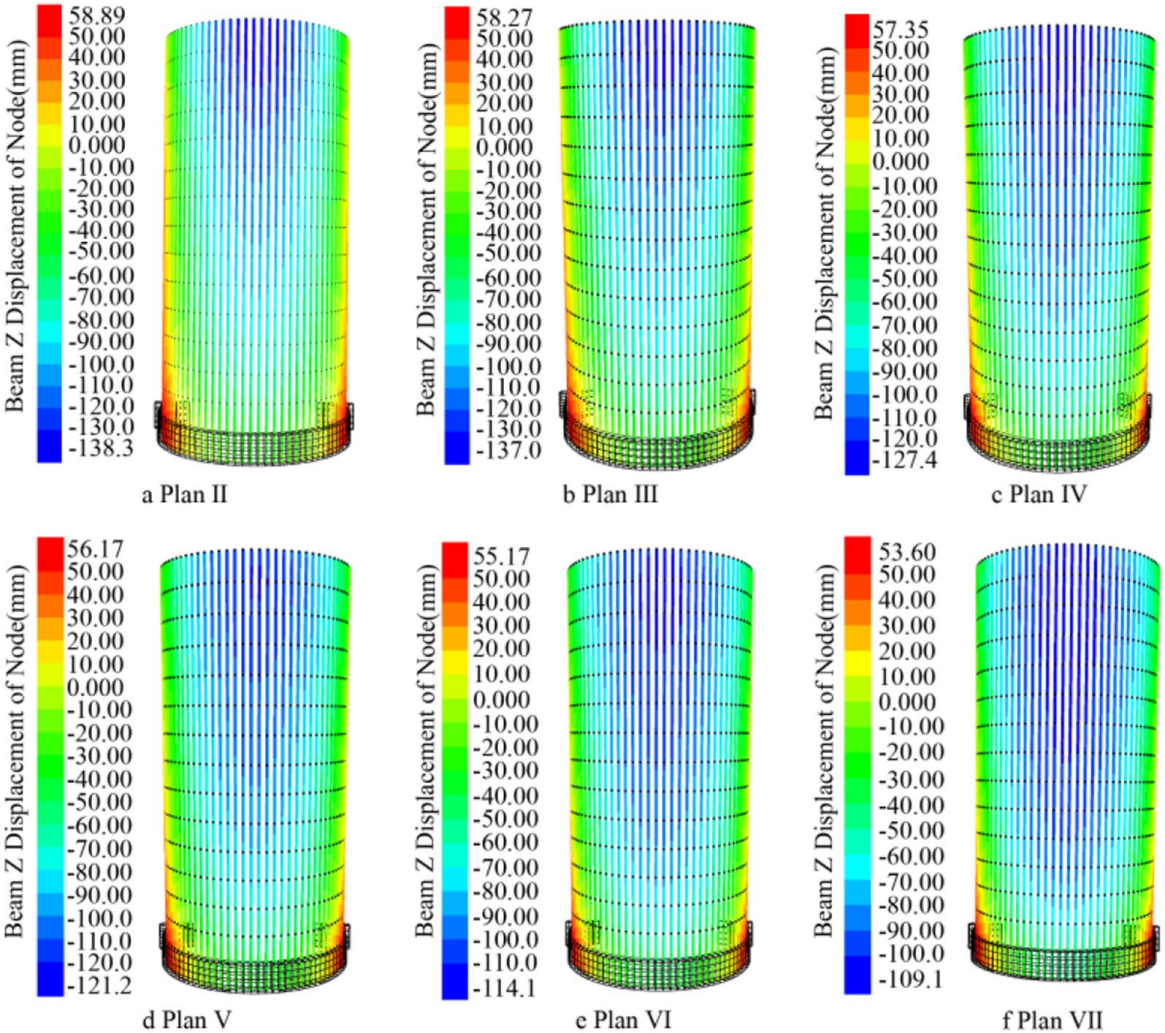
Displacement cloud of pipe shed.

When the excavation of section 15 (30m) is completed, the cloud map of pipe shed displacement is observed, and the influence of all excavation disturbances on the maximum displacement of pipe shed is analyzed, as shown in Fig. 13. Comparing the area of the subsidence area (blue) in Fig. 13, it can be found that from Plan II to Plan VII, the area of the subsidence area of the pipe shed gradually increases. Plan II has the smallest area of subsidence, and the dark blue area is mainly concentrated at the end of the pipe shed inserted into the vault. Plan VII has the largest area of subsidence, and the dark blue area is concentrated in the middle area of the vault. In all six schemes, the rise of the arch is mainly concentrated on the two sides of the arch, and the range is small.

### Contrast of metacarpal surface deformation

The pebble surrounding rock has the characteristics of loose geology, large stone spacing, and easy water seepage; According to the ADECO-RS approach, the arch effect of surrounding rock during tunnel excavation is prone to the collapse of surrounding rock and large deformation of the tunnel face. The core idea of the ADECO-RS approach is that the large deformation during tunnel excavation is mainly caused by the deformation of the pre-reserved core soil on the tunnel face^19,20^. This article is based on the theory of the ADECO-RS Approach and compares the deformation of the excavation face of seven construction schemes. The ZK8 + 840 (excavation depth of 10 m) and ZK8 + 850 (excavation depth of 20 m) sections are selected to analyze the extrusion deformation. The maximum displacement value generated by the face at the current depth of each excavation section (2 m) was recorded, and the supporting effects of seven different plans were compared.

**Figure 14:**
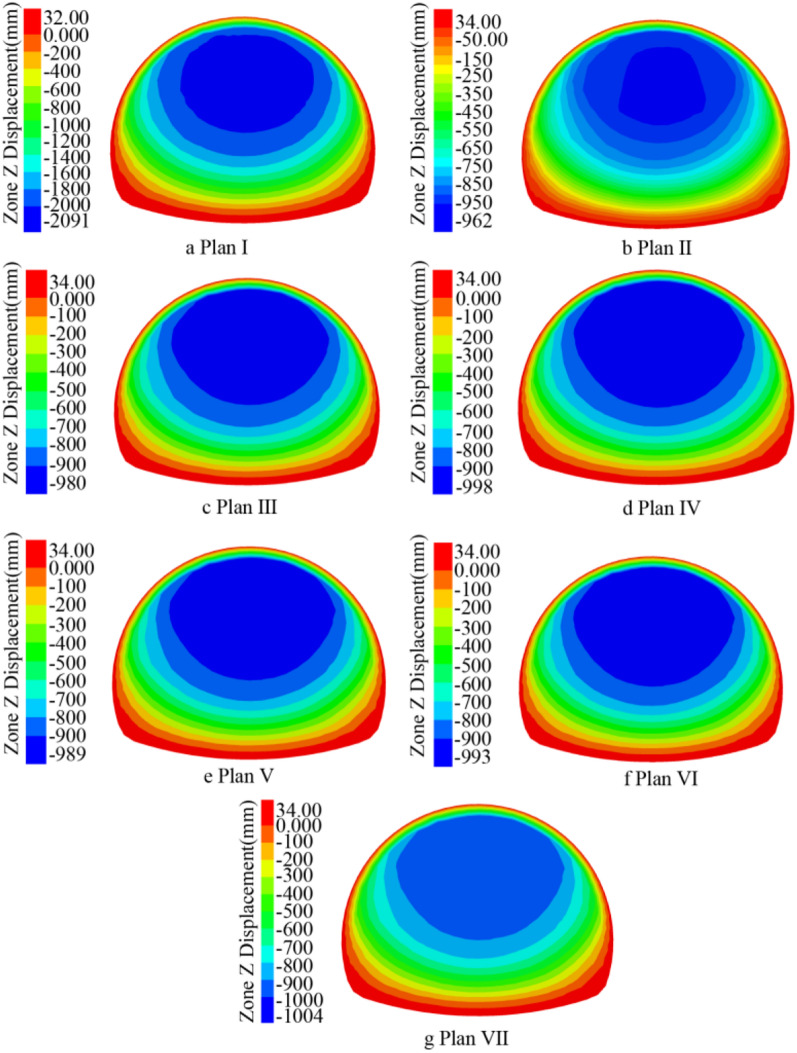
ZK8 + 840 palm surface displacement cloud map.

**Figure 15:**
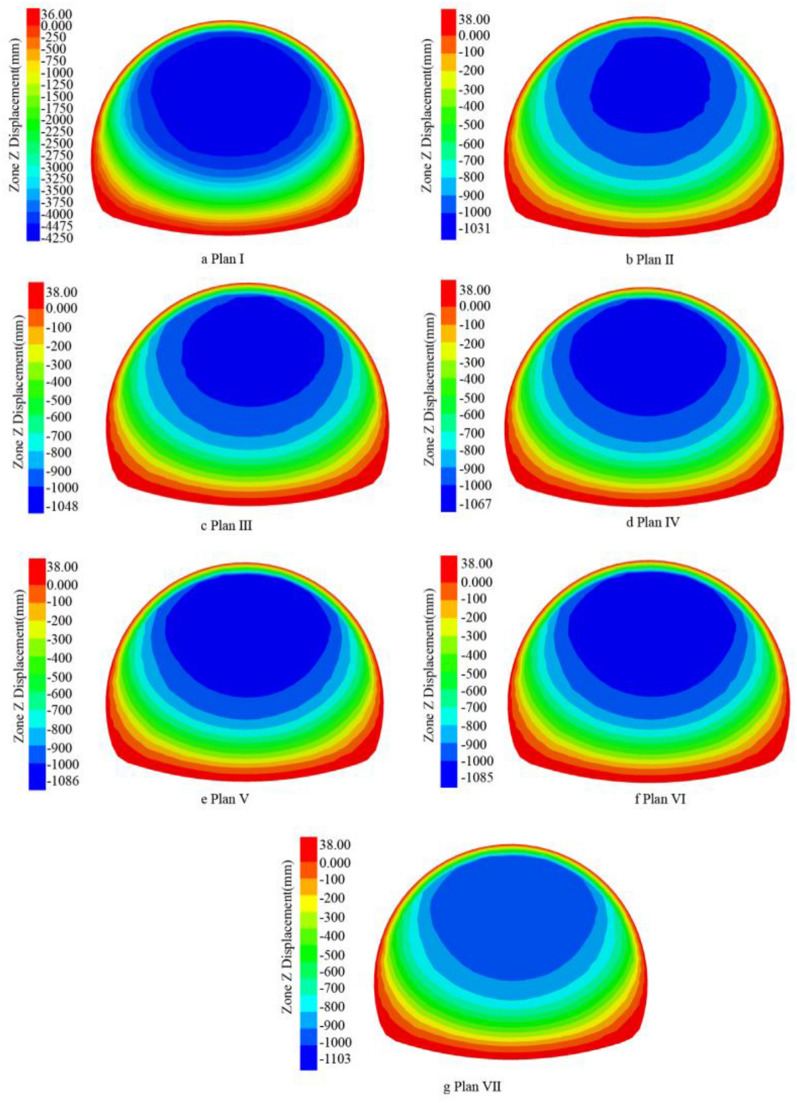
ZK8 + 850 palm surface displacement cloud map.

**Figure 16:**
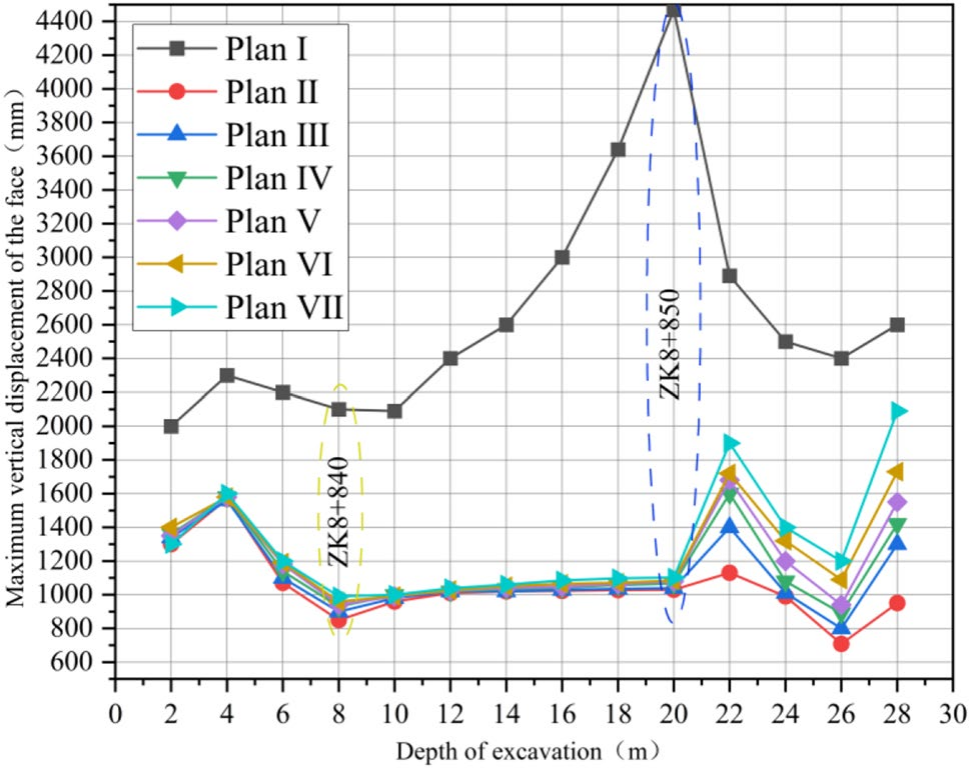
Vertical maximum displacement diagram of palm face.

Under different support plans, the displacement cloud map generated by the palm surface is obtained at the excavation depth of 10 m (Fig. 14, ZK8 + 840) and 20 m (Fig. 15, ZK8 + 850). Plan I has the largest vertical displacement. The vertical displacement value of the palm surface of Plan II to Plan VII gradually increases, and the maximum vertical displacement area (dark blue) gradually increases. By analyzing the maximum displacement value of the palm surface after each section of excavation (Fig. 16), it can be concluded that the grouting advance support of the long pipe shed has obvious effect on reducing the maximum vertical displacement of the palm surface, and different inclination angles have obvious influence on the palm surface displacement. At 10 m before the depth of excavation, different angles have little influence on the stability of the palm surface, and the data lines are basically in a state of coincidence. The influence of inclination angle on the palm surface increases gradually in the area of excavation depth of 10 m ~ 20 m, and the vertical displacement data lines of the palm surface gradually disperse. Each inclination angle in the 20 m ~ 30 m region has the greatest influence on the stability of the palm surface, and the data lines are obviously dispersed. From the perspective of vertical displacement value, the vertical displacement value of the palm face of plan VII is always greater than that of other inclination plans, and the vertical displacement value of the palm face gradually approaches that of plan I after 26 m depth, and the surface advance support gradually loses its supporting effect on surrounding rock. By comparing the numerical curves of plans II to VII in Fig. 16, the maximum displacement range generated by the palm surface is basically 1 m, which is due to the low cohesion of the pebble layer and the broken structure of the surrounding rock. When the excavation causes a large range of disturbance to the surrounding rock structure, the excavation face will produce large deformation. It is recommended to adopt the pre-reinforcement support scheme of the face to reduce the maximum displacement value of the face.

### Plastic zone range comparison

**Figure 17:**
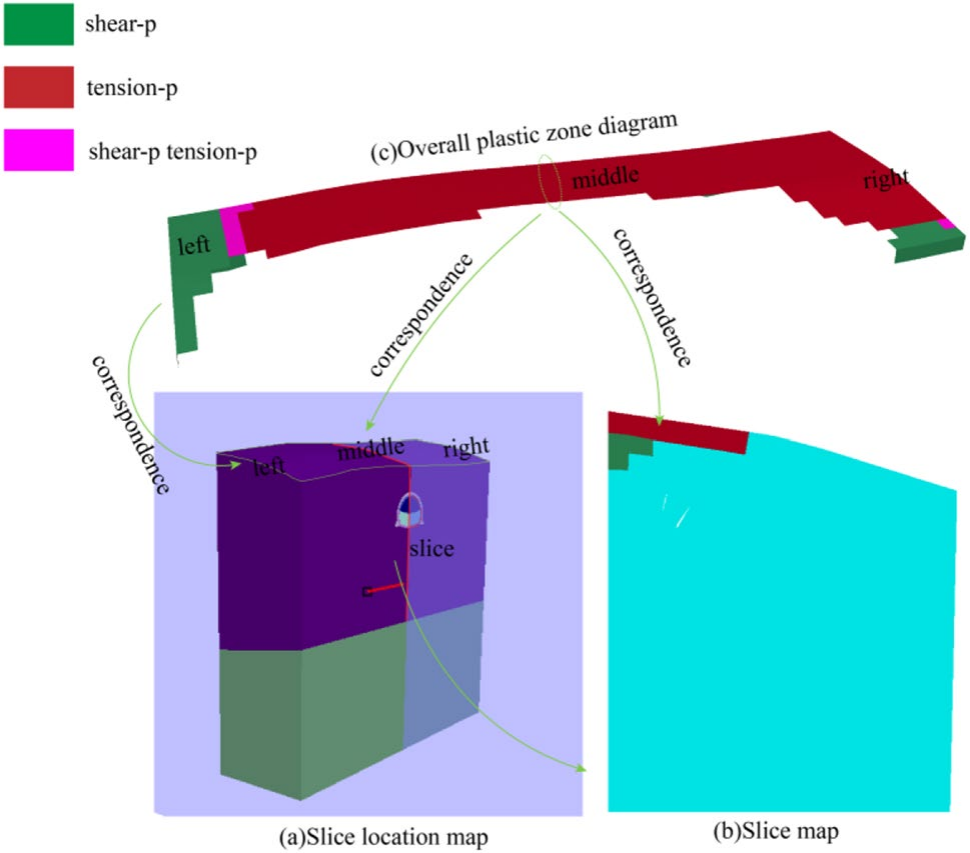
Natural state plastic zone distribution.

The distribution of surrounding rock plastic zone in tunnel excavation can be obtained by FLAC^3D^ numerical simulation. The larger the distribution range of surrounding rock plastic zone, the larger the shear deformation and tension deformation area of surrounding rock caused by excavation disturbance^21,22^. Figure 1 shows the plastic division layout of the research model under natural conditions. In order to facilitate the study of the disturbance effect of excavation on surrounding rock, the slicing tool is used to set the slice coordinates as (50, 15, 50) and the vector as (1, 0, 0) to obtain the plastic division layout of surrounding rock at the central axis of the tunnel. It can be observed from Fig. 17 that under natural conditions, shear and tension phenomena occur on the top surface (ground) of the pebble layer due to the influence of gravity field, but the overall distribution on the surface is small and the distribution depth is shallow, and there is no disturbance inside the pebble layer.

**Figure 18:**
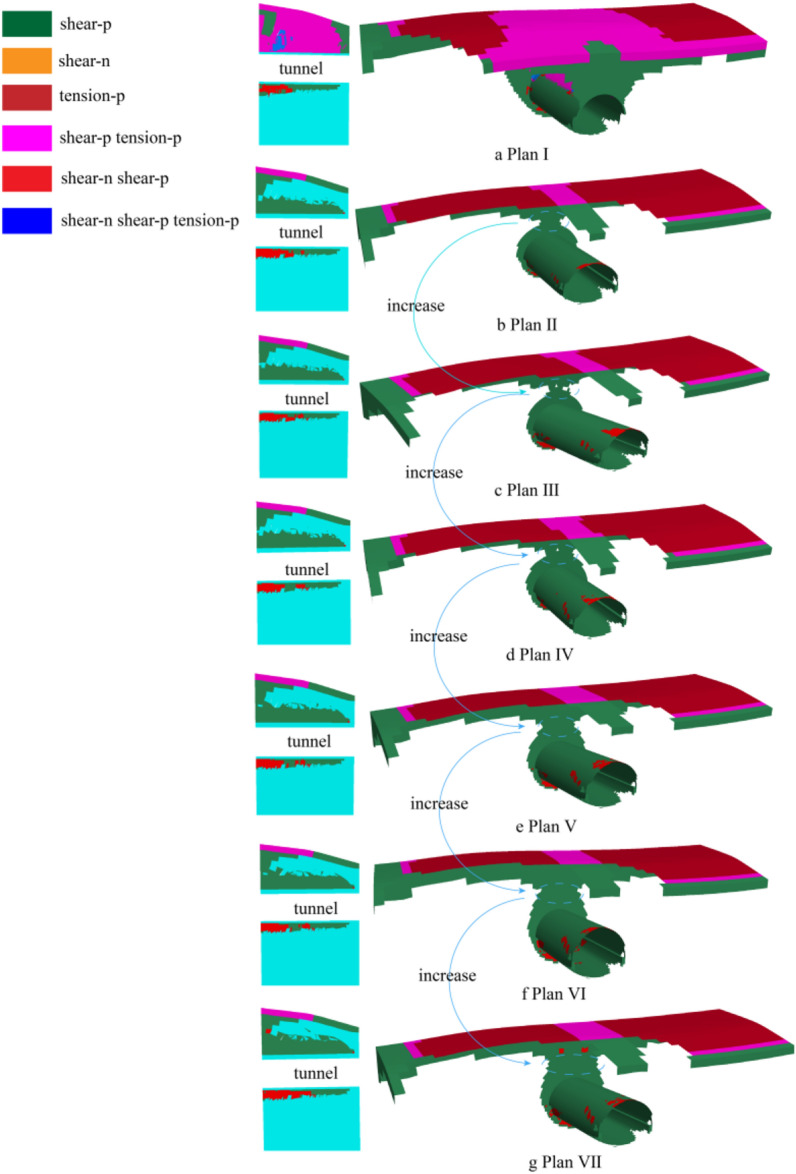
Tunnel excavation plastic zone distribution.

Compared with Figs. 17 and 18, it can be clearly observed that tunnel excavation increases the disturbance effect on the pebble layer, and the surrounding rock surrounding the tunnel contour produces more shear failure due to excavation, and some areas produce different degrees of tension failure. Compared with the seven research results in Fig. 18, the plastic zone range of surrounding rock caused by not using advance support excavation (Plan I) is the largest. According to the analysis of the section diagram of the central axis of the tunnel, the pebble layer above the tunnel in plan I has a large range of shear and tension failure, and the failure area extends to the ground of the pebble layer. According to the ADECO-RS Approach, the soil mass above the tunnel becomes unstable and collapse occurs in the process of tunnel excavation. The comparison between the other six plans and plan I shows that the advance support greatly reduces the disturbance effect on the pebble layer, and the pebble layer above the excavation entrance (ZK8 + 830) does not cause shear damage due to excavation. Moreover, it can be observed from the broken surface diagram that in the advanced support area, the pebble layer receives less disturbance, and the shear failure area is only in the tunnel contour attachment, and does not extend to the surface like plan I. However, in the second half of the excavation zone, the range of plastic zone produced under different advance support angles is quite different. According to the profile analysis of Fig. 18, the shear failure area of plan II is the smallest in pile No. ZK8 + 860, and the failure area of plan II to plan VII gradually increases. In plan VII, the plastic zone generated in part after tunnel excavation is penetrated to the surface, which is the same as plan I, and this part of the supporting area on the surface loses the advance supporting effect.

### Inclination affects geometric analysis

**Figure 19:**
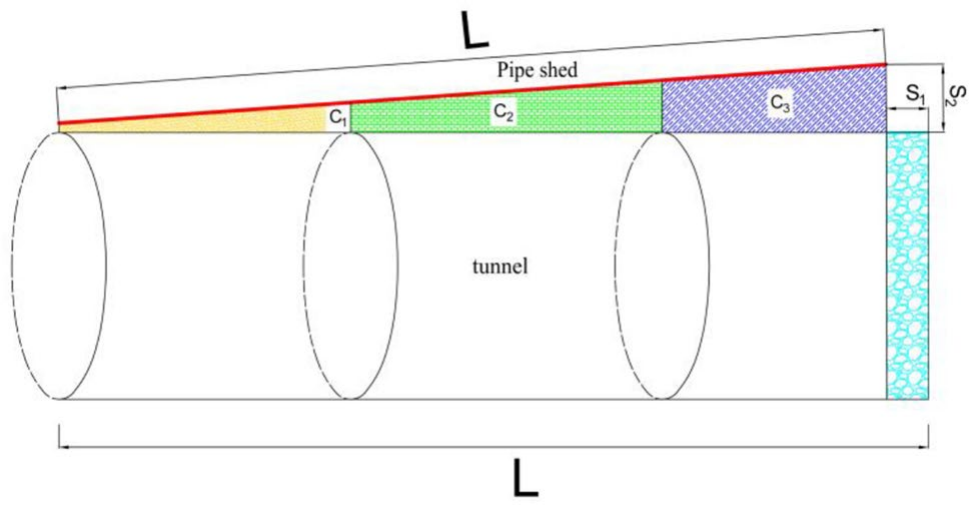
Pipe shed support diagram.

According to the triangular geometry principle, the dip angle will cause a plastic zone between the pipe shed and the tunnel contour, and the plastic zone range will increase with the increase of the dip angle. From the above analysis results, it can be concluded that every 10 m of the long pipe shed is a partition. In Fig. 19, the pipe shed with a length of 30 m is divided into three sections, and the plastic zone range is divided into C_1_, C_2_ and C_3_. In the range of C_1_ zone, the inclination angle has little influence on the surrounding rock structure, the difference of the structural stability in the plastic zone of C_2_ zone increases, and the difference of the structural stability in the plastic zone of C_3_ zone is obvious. According to the triangle theory, when the inclination angle increases, the S_2_ region scope increases, and the advance support effect on the surrounding rock in the tunnel contour weakens. Moreover, the range of S_1_ area (unsupported area) will gradually increase. According to the principle of new method, the stability of surrounding rock excavation in this section is poor, and it can be concluded from the comparison of the range of plastic zone that this section of rock mass excavation is prone to collapse hazards.

### Influence of inclination angle on force analysis

**Figure 20:**
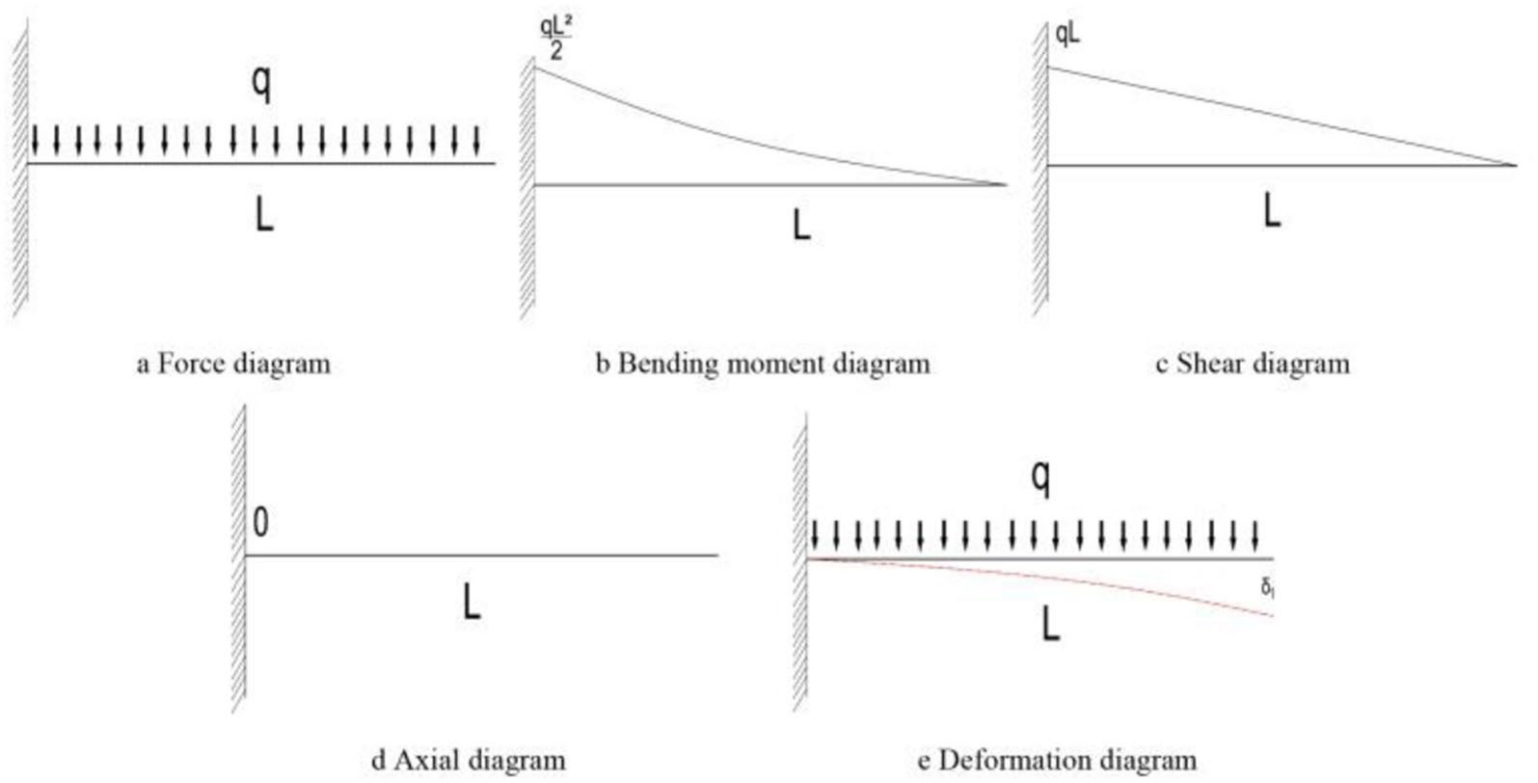
Mechanical analysis of pipe shed without offset angle.

**Figure 21:**
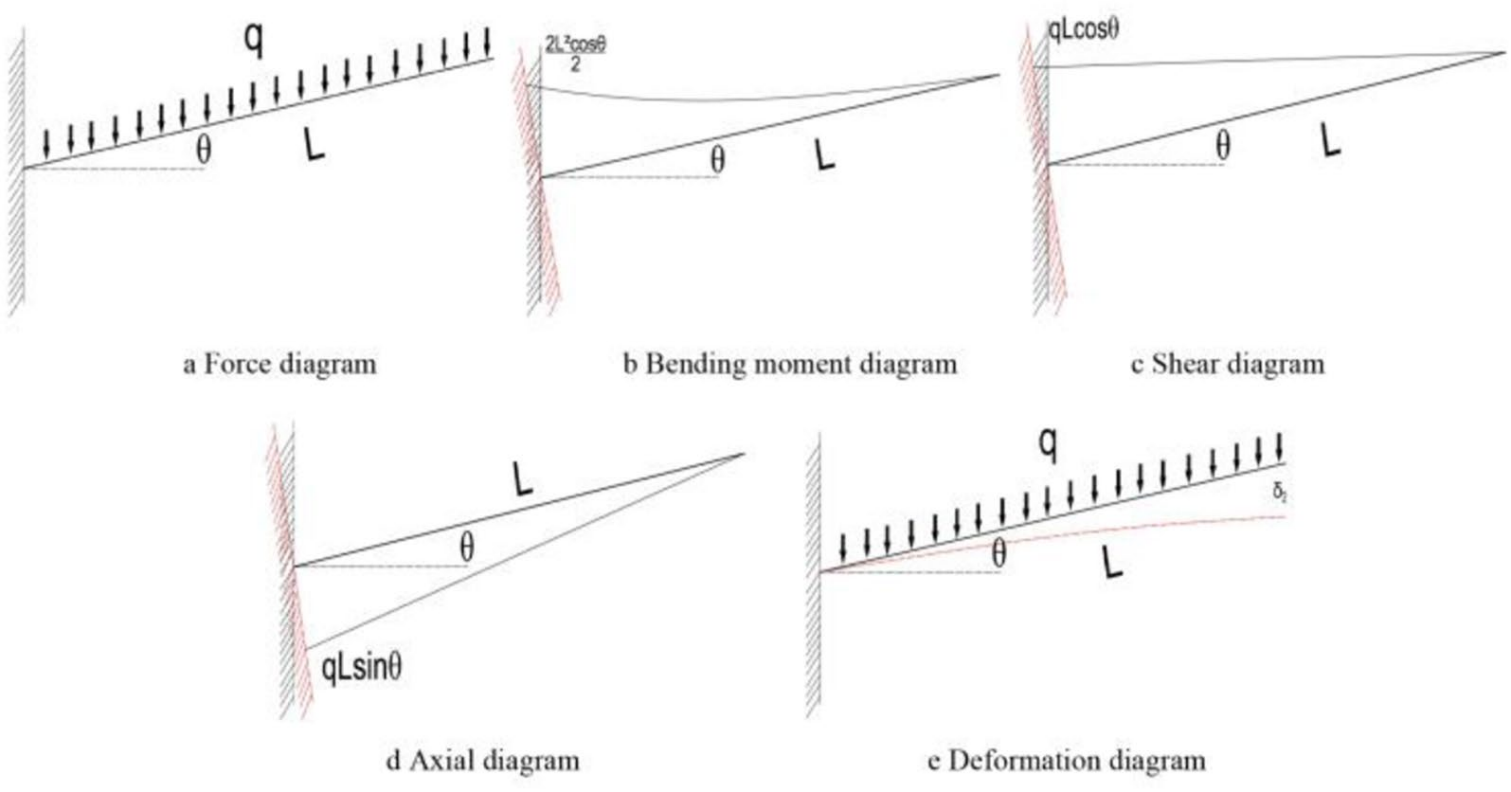
Mechanical analysis of deflected angle pipe shed.

One end of the shed is fixed in the set arch, and the other end can be moved freely, so the long pipe shed after construction is regarded as a cantilever beam. Vertical uniform load is distributed above the pipe shed. Force analysis is carried out on horizontal pipe shed and inclined pipe shed respectively. The analysis results are shown in Figs. 20 and 21.

According to the comparative force, analysis results in Figs. 20 and 21, it can be seen that; under vertical load, the bending moment and shear value of the pipe shed decrease with the increase of the dip angle, and the axial force value of the pipe shed increases with the increase of the dip angle. By comparing the deformation diagram of the pipe shed, it is found that the type variable of pipe shed with inclination angle under vertical load is smaller than that of horizontal pipe shed. The analysis result of the type variable is the same as the maximum vertical value of the pipe shed in Fig. 13.

## Comparative analysis of measured values

**Figure 22:**
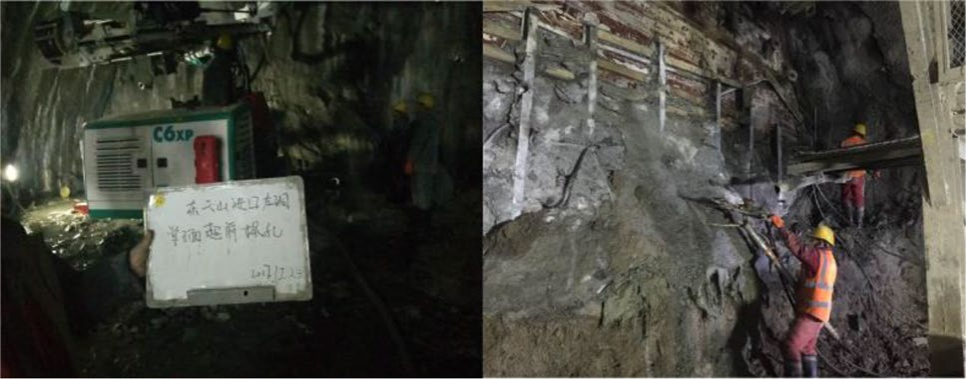
Hole excavation site diagram.

**Figure 23:**
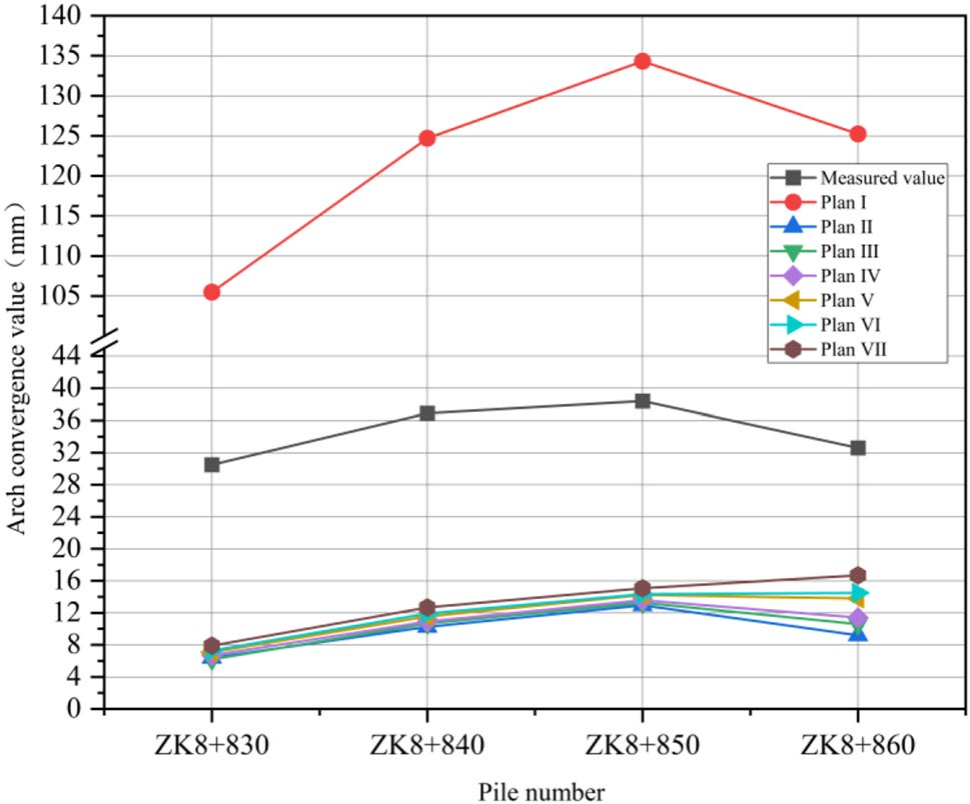
Comparison of the convergence values of the vault.

**Figure 24:**
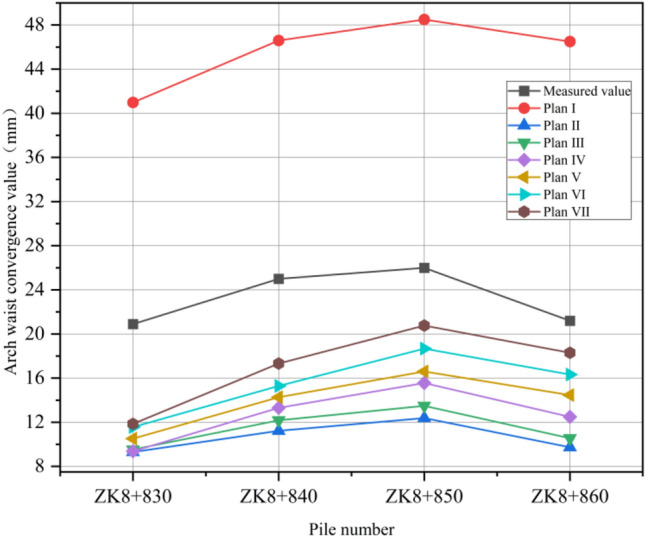
Comparison of convergence values of arch waist.

ZK8 + 830 ~ ZK8 + 860 is located in the pebble layer, the shallowest burial depth is only 7 m; before excavation, 108 × 6 mm hot-rolled seamless steel pipe is used for grouting reinforcement. The tunnel is excavated in full section with a depth of 2 m for each section. After the completion of excavation, C25 concrete was sprayed for the initial support, and the convergence change of surrounding rock was monitored by total station and displacement meter at the vault and waist of the tunnel. After the instrument is placed, special personnel are arranged to record the change of convergence value of surrounding rock every day. The construction site of the tunnel left line entrance is shown in Fig. 22. In order to verify the rationality of the numerical simulation, four excavation pile faces (ZK8 + 830, ZK8 + 840, ZK8 + 850, ZK8 + 860) were selected for analysis. Data monitoring was carried out on the four simulated pile faces, and the convergence values of the vault and arch were analyzed. The simulation values are compared with the measured data. The numerical comparison curves are shown in Figs. 23 and 24.

According to the comparative analysis of Figs. 23 and 24, the convergence value of surrounding rock is much higher than the measured data when the excavation of plan I is adopted, indicating that the instability of surrounding rock is easy to occur if the support reinforcement is not carried out in advance in shallow excavation of pebble layer. According to the trend of value change, the monitoring data of plan II to plan VII in ZK8 + 830 to ZK8 + 840 section have little difference, and the convergence value of surrounding rock is basically the same. When the pile surface of ZK8 + 850 is excavated from the back, the convergence value difference between the vault and the waistband increases, and the convergence value of plan VII is the largest, which is the same as the distribution range of the plastic zone. From the analysis of the convergence change of surrounding rock, the convergence curve of the arch and the arch waist of plan II is the most similar to the measured data curve, which indicates that the construction simulation of 0° inclination angle of pipe shed is consistent with the actual engineering excavation. Through comparison, it is concluded that using advance support scheme in pebble layer shallow buried tunnel excavation can effectively reduce the convergence value of surrounding rock and maintain the stability of surrounding rock. Therefore, in the process of tunnel construction, the excavation of shallow buried biased pressure tunnel entrance should be unified with support, and then monitoring and measurement should be carried out, and a large amount of density and frequency should be measured, and timely attention should be paid to the deformation of surrounding rock and lining to prevent engineering disasters. When the construction dip angle of the long pipe shed is large, the weakening effect of the increasing dip angle on the supporting effect should be paid attention to when the tunnel excavation enters the second half of the supporting area of the long pipe shed. In the area near the edge of the long pipe shed support, the palm surface should be strengthened in advance, and the small pipe should be strengthened in advance, so as to prevent the surrounding rock from large deformation and collapse due to the weakening of the advance support effect.

## Conclusion

1. Through the comparison of seven construction Plans and the theory of the ADECO-RS Approach, it is concluded that the long pipe shed advanced support can cement and solidify the loose rock mass, thus improving the mechanical properties of the rock mass and enhancing the bearing capacity of the surrounding rock. The long pipe shed advance support can play an important role in cushioning the loose step of the upper rock mass, preventing the subsidence of the upper loose rock mass, inhibiting the displacement of rock strata, and controlling the surface settlement. Through the comparison of the schemes, the displacement and deformation of the lining structure are small and the stress distribution is uniform when advanced support is used. The overall displacement area of the pipe shed, the convergence value of the face, and the range of the plastic zone of the surrounding rock are all far less than the simulation results without advanced support.
2. By comparing the vertical displacement of the pipe shed, the vertical displacement of the palm surface, and the distribution range of the plastic zone in the excavation process, it is concluded that the construction of a long pipe shed with different angles will produce a plastic zone of surrounding rock between pipe shed and vault; The difference of structural stability in the plastic zone of C_1_ zone is small, the difference of structural stability in the plastic zone of C_2_ zone is larger, and the difference of structural stability in the plastic zone of C_3_ zone is obvious. When the dip Angle is above 5°, the advance supporting effect of the surrounding rock in the C_3_ zone disappears.
3. According to the trigonometric theory, the size of S_1_ and S_2_ is related to the dip angle of the pipe shed. When the dip Angle is large, the surrounding rock supporting effect is greatly weakened when the increase of S_2_ area, and the arch effect is not easy to form after the excavation of the surrounding rock. The surrounding rock in S_1_ (cyan range) area fails to carry out advanced support, and the excavation is easy to cause collapse hazards. From the mechanical point of view, the bending moment, shear force, and shape variable of pipe shed decrease with the increase of dip angle, and the axial force increases with the increase of dip angle. According to the comparison results of Plan II to Plan VII, combined with the fitting effect analysis of the simulated value and the measured value, it is suggested that the construction inclination angle of the long pipe shed should be set within the range of 0° ~ 3°.

## Data Availability

Te datasets used and/or analysed during the current study available from the corresponding author on reasonable request.
